# Computational Prediction of the Protonation Sites of Ac-Lys-(Ala)*n*-Lys-NH2 Peptides through Conceptual DFT Descriptors

**DOI:** 10.3390/molecules22030458

**Published:** 2017-03-13

**Authors:** Sebastián Sastre, Juan Frau, Daniel Glossman-Mitnik

**Affiliations:** 1 Departament de Química, Universitat de les Illes Balears, 07122 Palma de Mallorca, Spain; sebastiansastre43@gmail.com (S.S.); juan.frau@uib.es (J.F.); 2 Laboratorio Virtual NANOCOSMOS, Centro de Investigación en Materiales Avanzados, Departamento de Medio Ambiente y Energía, Chihuahua, Chih 31136, Mexico

**Keywords:** diabetes, alzheimer, peptides, MEDT, conceptual DFT

## Abstract

Six density functionals (M11, M11L, MN12L, MN12SX, N12, and N12SX) in connection with the Def2TZVP basis set and the SMD solvation model (water as a solvent) have been assessed for the calculation of the molecular structure and properties of several peptides with the general formula Ac-Lys-(Ala)${}_{n}$-Lys-NH2, with *n* = 0 to 5. The chemical reactivity descriptors for these systems have been calculated through Conceptual density functional theory (DFT). The active sites for nucleophilic and electrophilic attacks have been chosen by relating them to the Fukui function indices, the condensed dual descriptor Δ*f*(r), and the electrophilic Parr functions. The results allowed the prediction of the protonation sites of the peptides and rendered a qualitative explanation of the difference in p*K*${}_{a}$ of the two Lys groups.

## 1. Introduction

Conceptual density functional theory (DFT, also known as chemical reactivity theory) is a powerful tool for the prediction, analysis, and interpretation of the outcome of chemical reactions [1,2,3,4].

Recently, Luis R. Domingo [5] proposed a new theory for the study of the reactivity in organic chemistry, which he named molecular electron density theory (MEDT). In this new theory, the capability for changes in electron density is responsible for the molecular reactivity [5], while the electron density distribution at the ground state is responsible for physical and chemical molecular properties.

Within MEDT (which encompasses conceptual DFT), several new concepts and reactivity descriptors have been defined, such as the global electron density transfer (GEDT), the nucleophilicity N index, and local condensed descriptors like the electrophilic *P*${}_{k}^{-}$ and nucleophilic *P*${}_{k}^{+}$ Parr functions [6]. The use of the Parr functions is becoming widespread for the understanding of organic chemistry processes, as evidenced by some recent publications related to Diels–Alder and cycloadditions reactions [7,8,9].

An interesting chemical reaction amenable to being studied through conceptual DFT is protein glycation. This reaction is initiated by a nucleophilic addition (nonenzymatic glycation or Maillard reaction), a reaction between a free amino group from a protein and a carbonyl group from a reducing sugar to form a freely reversible Schiff base. Glycated proteins can undergo further reactions, giving rise to some structures called advanced glycation endproducts (AGEs) [10].

Indeed, the glycation sites of an amino acid, peptide, or protein may also be the preferred sites for protonation. The reason for this assertion is because from a chemical point of view, the interaction of the carbon atom of a reducing carbonyl compound with an amino nitrogen atom is the same kind of reaction that occurs when a proton interacts with that nitrogen atom of the amino group. For a peptide or protein with many glycation or protonation sites, there is a rank of preferred reaction sites that depends on the pH, the solvent, and the conformational structure of the molecular system. This means that that if we know the ordering of the protonation of the peptide or protein, it will be possible to discern (at least qualitatively) the p*K*${}_{a}$ of the Lys residues.

In some recent interesting works [11,12], Makowska et al. studied the acidic–basic properties of alanine-based peptides containing acidic and basic side chains. In particular, they experimentally estimated the influence of the length of the alanine spacer on the p*K*${}_{a}$ of the Lys residues of Ac-Lys-(Ala)*n*-Lys-NH2 peptides (wth *n* = 0, 1, 2, *…*, 5) [11].

Following the pioneering work of Parr et al. [1], a number of useful concepts have been derived from the analysis of the density of any molecular system through DFT. These concepts that allow a researcher to make qualitative predictions about the chemical reactivity of a given system can also be quantified, and are collectively known as conceptual DFT Descriptors.

Therefore, we believe that it could be of interest to apply the concepts of density functional theory to the study of those peptides in order to determine if it is possible to discern between the potential protonation sites and to obtain a qualitative estimation of the p*K*${}_{a}$ differences between them.

In order to obtain quantitative values of the conceptual DFT descriptors, it is necessary to resort to the Kohn–Sham theory through calculations of the molecular density, the energy of the system, and the orbital energies—specifically those related to the frontier orbitals; that is, HOMO and LUMO [13,14,15,16,17,18].

Although the foundations of DFT have established that a universal density functional must exist and that all of the properties of the system can be obtained through calculations with this functional, in practice one must resort to some of the approximate density functionals that have been developed in the last thirty years. Because these are approximate functionals (i.e., not universal functionals), many of them are good for predicting some properties and others are good for other properties. Sometimes you can find density functionals that are excellent for describing the properties of a given molecular system with a particular functional group, but it is necessary to resort to other density functionals for a different functional group that you want to include in the molecular system under study.

When one is dealing with the study of chemical reactivity (i.e., a process that involves the transference of electrons), it is normal to perform calculations not only of the ground state, but also for open systems like the radical cation and radical anion. These systems are often difficult to converge giving trustworthy results—specially if diffuse functions must be included in the basis set [13,14,15,16,17,18]. For this reason, it is convenient to have a method that can give all information that one needs directly from the results of the calculation of the ground state of the molecular system under study. In particular, one may want to obtain the ionization potential (I) and electron affinity (A) of the system avoiding the calculation of the anion and cation radicals. Indeed, the link for this is given by the so-called Koopmans’ theorem [15,16,17,18].

Koopmans’ theorem is not valid within DFT. However, from an empirical and practical point of view, it is meaningful to follow the procedure of assigning KS HOMO as equal to and opposite of the vertical ionization potential, $\epsilon_{H}$ = –I, and the KS LUMO as equal to and opposite of the vertical electron affinity, $\epsilon_{L}$ = –A. We have coined the acronym KID (“Koopmans in DFT”) for this empirical procedure.

This means that the goodness of a given density functional for the purpose of predicting the Conceptual DFT descriptors directly from the properties of the neutral molecule can be estimated by checking how well it follows the KID procedure. Thus, it will be interesting to consider several recent density functionals that have shown great accuracy across a broad spectrum of databases in chemistry and physics [19] to evaluate their performance in fulfilling this practical technique.

The objective of this work is twofold: (i) to conduct a comparative study of the performance of some density functionals from the Minnesota family for reproducing chemical reactivity descriptors of the Ala-spaced Lys-containing peptides proposed by Makowska et al. [12] within the KID formalism; and (ii) to predict the preferred protonation sites that could be give a qualitative estimation of the p*K*${}_{a}$ of the Lys residues using condensed dual descriptors.

## 2. Theoretical Background

As this work is part of an ongoing project, the theoretical background is similar to that presented in previous research [20,21,22,23,24,25,26,27] and will be shown here for the sake of completeness. Within the conceptual framework of DFT [2,28], the chemical potential *μ* is defined as:

(1) $$\mu ={\left(\frac{\partial E}{\partial N}\right)}_{v(\vec{r})}=-\chi \;,$$

where *χ* is the electronegativity, while the global hardness *η* is

(2) $$\eta ={\left(\frac{\partial^{2}E}{\partial N^{2}}\right)}_{v(\vec{r})}\;.$$

Using a finite difference approximation and the KID procedure, the above expressions can be written as:

(3) $$\mu =-\frac{1}{2}\left(I+A\right)\approx \frac{1}{2}\left(\epsilon_{L}+\epsilon_{H}\right)=\chi_{K}\;,$$

(4) $$\eta =\left(I-A\right)\approx \left(\epsilon_{L}-\epsilon_{H}\right)=\eta_{K}\;,$$

where $\epsilon_{H}$ and $\epsilon_{L}$ are the energies of the highest occupied and the lowest unoccupied molecular orbitals (HOMO and LUMO), respectively. The electrophilicity index *ω* has been defined as [29]:

(5) $$\omega =\frac{\mu^{2}}{2\eta }=\frac{{\left(I+A\right)}^{2}}{4(I-A)}\approx \frac{{\left(\epsilon_{L}+\epsilon_{H}\right)}^{2}}{4(\epsilon_{L}-\epsilon_{H})}=\omega_{K}\;.$$

The Fukui function is defined in terms of the derivative of ρ(r) with respect to *N* [2]:

(6) $$f\left(r\right)={\left(\frac{\partial \;\rho (r)}{\partial \;N}\right)}_{\upsilon (r)}$$

The function f(r) reflects the ability of a molecular site to accept or donate electrons. High values of f(r) are related to a high reactivity at point r [2]. By applying a finite difference approximation to Equation (6), two definitions of Fukui functions depending on total electronic densities are obtained:

(7) $$\begin{array}{ccc} f^{+}\left(r\right) & = & \rho_{N+1}\left(r\right)-\rho_{N}\left(r\right)\;, \end{array}$$

(8) $$\begin{array}{ccc} f^{-}\left(r\right) & = & \rho_{N}\left(r\right)-\rho_{N-1}\left(r\right)\;, \end{array}$$

where $\rho_{N+1}\left(r\right)$, $\rho_{N}\left(r\right)$, and $\rho_{N-1}\left(r\right)$ are the electronic densities at point r for the system with N+1, *N*, and N-1 electrons, respectively. The first one, $f^{+}\left(r\right)$, has been associated to reactivity for a nucleophilic attack, so it measures the intramolecular reactivity at the site r towards a nucleophilic reagent. The second one, $f^{-}\left(r\right)$, has been associated to reactivity for an electrophilic attack, so this function measures the intramolecular reactivity at the site r toward an electrophilic reagent [28].

Morell et al. [3,30,31,32,33,34,35] proposed a local reactivity descriptor (LRD) which is called the dual descriptor (DD) $f^{\left(2\right)}\left(r\right)\equiv ∆f\left(r\right)$. The definition of ∆f(r) is shown as indicated by Morell et al. [30,31]:

(9) $$∆f\left(r\right)={\left(\frac{\partial \;f(r)}{\partial \;N}\right)}_{\upsilon (r)}\;.$$

The dual descriptor can be condensed over the atomic sites: when $∆f_{k}>0$, the process is driven by a nucleophilic attack on atom *k*, and then that atom acts as an electrophilic species; conversely, when $∆f_{k}<0$, the process is driven by an electrophilic attack over atom *k*, and therefore atom *k* acts as a nucleophilic species.

In an interesting and very enlightening article [36], Jorge Ignacio Martínez-Araya has shown why the dual descriptor is a more accurate local reactivity descriptor than the Fukui functions. He has shown that although the Fukui function has the capability of revealing nucleophilic and electrophilic regions on a molecule, the dual descriptor is able to unambiguously expose truly nucleophilic and electrophilic regions, but along with the latter, dual descriptor is less affected by the lack of relaxation terms than the Fukui function when the frontier molecular orbital approximation is applied, and that this implies that the dual descriptor can be considered a more reliable descriptor to measure local reactivity than Fukui function. For this reason, we have chosen the DD to fulfil the objectives of our study instead of the Fukui functions individually.

In 2013, Domingo proposed the Parr functions *P*(r) [37,38], which are given by the following equations:

(10) $$P{}^{-}\left(r\right)=\rho_{s}^{rc}\left(r\right)\;\;\;\;\;\;\left(\mathrm{for}\;\mathrm{electrophilic}\;\mathrm{attacks}\right),$$

(11) $$P{}^{+}\left(r\right)=\rho_{s}^{ra}\left(r\right)\;\;\;\;\;\;\left(\mathrm{for}\;\mathrm{nucleophilic}\;\mathrm{attacks}\right),$$

which are related to the atomic spin density (ASD) at the **r** atom of the radical cation or anion of a given molecule, respectively. The ASD over each atom of the radical cation and radical anion of the molecule gives the local nucleophilic *P*${}_{k}^{-}$ and electrophilic *P*${}_{k}^{+}$ Parr functions of the neutral molecule [6].

## 3. Settings and Computational Methods

Following the lines of our previous work [20,21,22,23,24,25,26,27], the computational studies were performed with the Gaussian 09 [39] series of programs with density functional methods as implemented in the computational package. The equilibrium geometries of the molecules were determined by means of the gradient technique. The force constants and vibrational frequencies were determined by computing analytical frequencies on the stationary points obtained after the optimization to check if there were true minima. The basis set used in this work was Def2SVP for geometry optimization and frequencies, while Def2TZVP was considered for the calculation of the electronic properties [40,41].

For the calculation of the molecular structure and properties of the studied systems, we chose six density functionals from the latest Minnesota density functionals family which consistently provide satisfactory results for several structural and thermodynamic properties [19]: M11, which is a is a range-separated hybrid meta-GGA (generalized gradient approximation) [42]; M11L, which is a dual-range local meta-GGA [43]; MN12L, which is a nonseparable local meta-NGA (nonseparable gradient approximation) [44]; MN12SX, which is a range-separated hybrid nonseparable meta-NGA [45]; N12, which is a nonseparable gradient approximation [46]; and N12SX, which is a range-separated hybrid nonseparable gradient approximation [45]. In these functionals, GGA stands for generalized gradient approximation (in which the density functional depends on the up and down spin densities and their reduced gradient) and NGA stands for nonseparable gradient approximation (in which the density functional depends on the up/down spin densities and their reduced gradient, and also adopts a nonseparable form). All the calculations were performed in the presence of water as a solvent by doing Integral Equation Formalism–Polarized Continuum Model (IEF-PCM) computations according to the solvation model density (SMD) solvation model [47].

## 4. Results and Discussion

**Figure 1:**
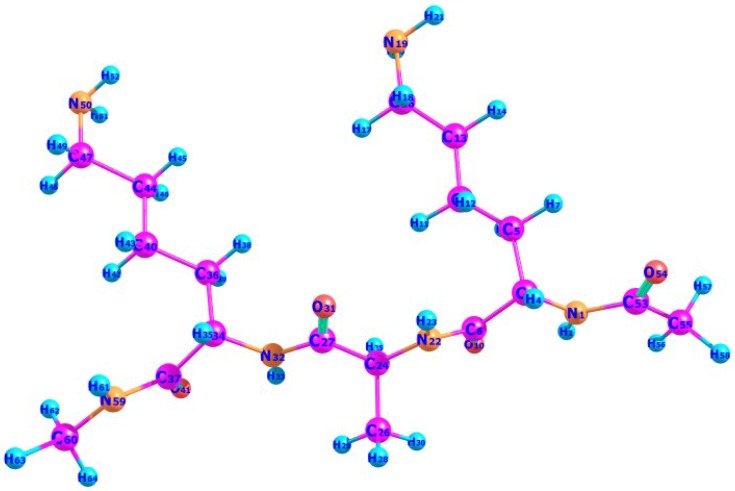
Geometrical conformation for the KAK peptide(Ac-Lys-(Ala)${}_{n}$-Lys-NH2 with n= 1).

The molecular structures of the six peptides with the general formula Ac-Lys-(Ala)${}_{n}$-Lys-NH2, with n= 0 to 5 were built by means of the Avogadro 1.2.0 program [48,49]. The resulting structures were pre-optimized by finding the most stable conformers through a random sampling with molecular mechanics techniques and a consideration of all the torsional angles through a random sampling with molecular mechanics techniques and a consideration of all the torsional angles through the general AMBER force field [50]. The structures of the resulting conformers were then reoptimized with the six density functionals mentioned in the previous section in conjunction with the Def2SVP basis set and the SMD solvation model, using water as a solvent. As an illustration of the optimized peptides, the geometrical conformation of Ac-Lys-(Ala)${}_{n}$-Lys-NH2 with n= 1 (KAK) is presented in Figure 1.

The HOMO and LUMO orbital energies (in eV), vertical ionization potentials I, and electron affinities A (in eV), global electronegativity *χ*, total chemical hardness *η*, and global electrophilicity *ω* of the peptides calculated with the six density functionals and the Def2TZVP basis set using water as as solvent simulated with the SMD parametrization of the IEF-PCM model are presented in Tables S1A to S6A of the Electronic Supplementary Information (ESI). The upper part of the tables shows the results derived assuming the KID procedure (hence the subscript K) and the lower part shows the results derived from the calculated ΔSCF vertical energies.

With the objective of analyzing our results in order to verify the fulfillment of the KID procedure, we previously designed several descriptors that relate the results obtained through the HOMO and LUMO calculations with those obtained by means of the vertical I and A with a ΔSCF procedure. The first three descriptors are related to the simplest fulfillment of Koopmans’ theorem by relating $\epsilon_{H}$ with –I, $\epsilon_{L}$ with –A, and the behavior of them in the description of the band gap: $J_{I}=\left|\epsilon_{H}+E_{gs}\left(N-1\right)-E_{gs}\left(N\right)\right|$, $J_{A}=\left|\epsilon_{L}+E_{gs}\left(N\right)-E_{gs}\left(N+1\right)\right|$, and $J_{HL}=\sqrt{{J_{I}}^{2}+{J_{A}}^{2}}$.

Next, we consider four other descriptors that analyze how well the studied density functionals are useful for the prediction of the electronegativity *χ*, the global hardness *η*, and the global electrophilicity *ω*, and for a combination of these conceptual DFT descriptors, considering only the energies of the HOMO and LUMO or the vertical I and A: $J_{\chi }=\left|\chi -\chi_{K}\right|$, $J_{\eta }=\left|\eta -\eta_{K}\right|$, $J_{\omega }=\left|\omega -\omega_{K}\right|$, and $J_{D1}=\sqrt{J_{\chi }^{2}+J_{\eta }^{2}+J_{\omega }^{2}}$, where D1 stands for the first group of conceptual DFT descriptors.

The results of the calculations of $J_{I}$, $J_{A}$, $J_{HL}$, $J_{\chi }$, $J_{\eta }$, $J_{\omega }$, and $J_{D1}$ for the six peptides considered in this work are displayed in Tables S1B to S6B of the Electronic Supplementary Information (ESI).

Based on the results for the descriptors presented on Tables S1B to S6B of the ESI, we have compiled the average values for each density functional on the whole group of peptides, and the calculated results are displayed in Table 1.

As can be seen from Table 1, the KID procedure holds with great accuracy for the MN12SX and N12SX density functionals, which are range-separated hybrid meta-NGA and range-separated hybrid NGA density functionals, respectively. Indeed, the values of *J*${}_{I}$, *J*${}_{A}$, and *J*${}_{HL}$ are not exactly zero. However, the results are impressive, particularly for the N12SX density functional.

It is interesting to see that the same density functionals also fulfill the KID procedure for the other descriptors—namely $J_{\chi }$, $J_{\eta }$, $J_{\omega }$, and $J_{D1}$. These results are very important, because they show that it is not enough to rely only on $J_{I}$, $J_{A}$, and $J_{HL}$. For example, if we consider only $J_{\chi }$, for all of the density functionals studied in this work, the values are very close to zero. As for the other descriptors, only the MN12SX and N12SX density functionals show this behavior. This means that the results for *J*${}_{\chi }$ could be due to a fortuitous cancellation of errors.

An important fact is that although the range-separated hybrid NGA and range-separated hybrid meta-NGA density functionals can be useful for the calculation of the conceptual DFT descriptors, it is not the same for the range-separated hybrid GGA (M11) density functional. An inspection of Table S1A of the ESI shows that this is due to the fact that this functional inadequately describes the energy of the LUMO, leading to negative values of A, which are in contradiction with the ΔSCF results.

The condensed Fukui functions and condensed dual descriptor have been calculated using the AOMix molecular analysis program [51,52] starting from single-point energy calculations.

The condensed dual descriptors $∆f_{k}$ and the electrophilic Parr functions *P*${}_{k}^{-}$(mpa) and *P*${}_{k}^{-}$(mpa) over the N atoms of the Lys residues of the KK, KAK, KA2K, KA3K, KA4K, and KA5K peptides of the Lys residues of the KK, KAK, KA2K, KA3K, KA4K, and KA5K peptides calculated with the MN12SX and N12SX density functionals and the Def2TZVP basis set using water as as solvent simulated with the SMD parametrization of the IEF-PCM model are shown in Table 2.

As the studied peptides are end-capped, it would be expected that the chemical reactivities of the Lys nitrogen atoms will be different. In fact, the values presented in Table 2 correspond to the condensed dual descriptors and electrophilic Parr functions over the nitrogen atoms of the Lys closer to the end-terminal Ac- group (Lys1). The values of the descriptors over the nitrogen atom of the other Lys residue (Lys2) are not shown because they are almost zero in all cases. This means that the preferred glycation site will be Lys1, but it will also be the preferred protonation site. Therefore, it can be predicted that the p*K*${}_{a}$ of Lys 1 will be greater than the p*K*${}_{a}$ of Lys2, although it is not possible to assign a particular value to both p*K*${}_{a}$s. Moreover, although there is not a clear trend, it can be observed from the results in Table 2 for the condensed dual descriptor and electrophilic Parr functions (either from a Mulliken population analysis (MPA) or Hirshfeld population analysis (HPA)) that the value of the descriptors are larger for KA4K and KA5K than for the other peptides. Thus, it can be concluded that the effect of the alanine spacers will be that of increasing the difference between the p*K*${}_{a}$ of Lys1 and and that of Lys2. This can probably be explained by considering that the increase of the number of Ala units leads to geometrical conformations of the peptides, making the terminal Lys residues be apart from each other and avoiding the competition between them for the reacting proton H${}^{+}$.

## 5. Conclusions

The Minnesota family of density functionals (M11, M11L, MN12L, MN12SX, N12, N12SX) has been tested for the fulfillment of the empirical KID procedure by comparison of the HOMO- and LUMO-derived values with those obtained through a ΔSCF technique. It has been shown that the range-separated hybrid meta-NGA density functional (MN12SX) and the range-separated hybrid NGA density functional (N12SX) are the best for the accomplishment of this objective. As such, they are a good alternative to those density functionals whose behavior have been tuned through a gap-fitting procedure and a good prospect for their usefulness in the description of the chemical reactivity of molecular systems of large size.

From the whole of the results presented in this work, it can be seen that the sites of interaction of the studied peptides can be predicted by using DFT-based reactivity descriptors such as the condensed dual descriptor and Parr functions calculations. These descriptors were used in the characterization and successful description of the preferred reactive sites and provide a firm explanation for the reactivity of those molecules.

Moreover, the preferred glycation and protonation sites could be identified, and helped to understand the effect of the alanine spacer on such properties as the p*K*${}_{a}$ of the different Lys residues. As a result, the difference between the p*K*${}_{a}$ of the Lys residue closer to the Ac- end-capped site and the p*K*${}_{a}$ of the other Lys residue will be always greater than zero, and will be increased as the number of alanine spacers grows.

## Figures and Tables

**Table 1:** Average descriptors *J*${}_{I}$, *J*${}_{A}$, *J*${}_{HL}$, *J*${}_{\chi }$, *J*${}_{\eta }$, *J*${}_{\omega }$, *J*${}_{D1}$ for the KK, KAK, KA2K, KA3K, KA4K and KAK5 peptides calculated with the M11, M11L, MN12L, MN12SX, N12, and N12SX density functionals and the Def2TZVP basis set using water as as solvent simulated with the solvation model density (SMD) parametrization of the Integral Equation Formalism–Polarized Continuum Model (IEF-PCM) model.

	*J*${}_{I}$	*J*${}_{A}$	*J*${}_{\mathrm{HL}}$	*J*${}_{\chi }$	*J*${}_{\eta }$	*J*${}_{\omega }$	*J*${}_{D1}$
M11	2.79	2.38	3.67	0.20	5.37	0.37	5.19
M11L	1.66	0.07	1.67	0.80	1.73	0.17	1.91
MN12L	1.87	0.14	1.88	0.87	2.01	0.8	2.20
MN12SX	0.13	0.08	0.15	0.09	0.06	0.05	0.14
N12	1.40	0.22	1.44	0.59	1.62	0.10	1.74
N12SX	0.00	0.07	0.07	0.03	0.07	0.03	0.08

**Table 2:** Condensed dual descriptors and electrophilic Parr functions *P*${}_{k}^{-}$ for the KK, KAK, KA2K, KA3K, KA4K, and KA5K peptides calculated with the MN12SX and N12SX density functionals and the Def2TZVP basis set using water as as solvent simulated with the SMD parametrization of the IEF-PCM model. MPA: Mulliken Population Analysis; HPA: Hirshfeld Population Analysis.

	MN12SX			N12SX		
	$∆f_{k}$	*P*${}_{k}^{-}$(mpa)	*P*${}_{k}^{-}$(hpa)	$∆f_{k}$	*P*${}_{k}^{-}$(mpa)	*P*${}_{k}^{-}$(hpa)
KK	−0.63	0.76	0.74	−0.69	0.76	0.73
KAK	−0.62	0.76	0.73	−0.69	0.76	0.73
KA2K	−0.71	0.81	0.78	−0.74	0.78	0.73
KA3K	−0.63	0.76	0.74	−0.69	0.76	0.73
KA4K	−0.71	0.81	0.78	−0.74	0.81	0.78
KA5K	−0.71	0.81	0.78	−0.74	0.81	0.78

## Supplementary Materials

The following are available online. ESI.pdf : Electronic Supplementary Information.

## Abbreviations

The following abbreviations are used in this manuscript:

DFT	Density Functional Theory
KS	Kohn-Sham
E	Electronic Energy
I	Ionization Potential
A	Electron Affinity
N	Number of Electrons
AGE	Advanced Glycation Endproducts
MEDT	Molecular Electron Density Theory
SCF	Self Consistent Field
KID	Koopmans in DFT
HOMO	Higher Ocuppied Molecular Orbital
LUMO	Lower Unocuppied Molecular Orbital
IEF-PCM	Integral Equation Formalism–Polarized Continuum Model
SMD	Solvation Model Density

## References

[1] Parr R., Yang W. (1989). Density-Functional Theory of Atoms and Molecules.

[2] Geerlings P., de Proft F., Langenaeker W. (2003). Conceptual Density Functional Theory. Chem. Rev..

[3] Toro-Labbé A. (2007). Theoretical Aspects of Chemical Reactivity.

[4] Chattaraj P. (2009). Chemical Reactivity Theory—A Density Functional View.

[5] Domingo L.R. (2016). Molecular Electron Density Theory: A Modern View of Reactivity in Organic Chemistry. Molecules.

[6] Domingo L.R., Ríos-Gutiérrez M., Pérez P. (2016). Applications of the Conceptual Density Functional Theory Indices to Organic Chemistry Reactivity. Molecules.

[7] Jasinski R. (2014). Searching for Zwitterionic Intermediates in Hetero Diels-Alder Reactions between Methyl *α*,p-dinitrocinnamate and Vinyl-Alkyl Ethers. Comput. Theor. Chem..

[8] Jasiński R., Mróz K., Kacka A. (2016). Experimental and Theoretical DFT Study on Synthesis of Sterically Crowded 2,3,3,(4)5-Tetrasubstituted-4-nitroisoxazolidines via 1,3-Dipolar Cycloaddition Reactions Between Ketonitrones and Conjugated Nitroalkenes. J. Heterocycl. Chem..

[9] Jasiński R., Jasińska E., Dresler E. (2017). A DFT Computational Study of the Molecular Mechanism of [3+2] Cycloaddition Reactions between Nitroethene and Benzonitrile N-oxides. J. Mol. Mod..

[10] Ahmed N. (2005). Advanced Glycation Endproducts—Role in Pathology of Diabetic Complications. Diabetes Res. Clin. Pract..

[11] Makowska J., Bagińska K., Liwo A., Chmurzynski L., Scheraga H. (2008). Acidic-Basic Properties of Three Alanine-Based Peptides Containing Acidic and Basic Side Chains: Comparison between Theory and Experiment. Biopolymers.

[12] Makowska J., Liwo A., Chmurzynski L., Scheraga H.A. (2012). Influence of the Length of the Alanine Spacer on the Acidic-Basic Properties of the Ac-Lys-(Ala)_*n*_-Lys-NH2 Peptides (*n* = 0, 1, 2, *…*, 5). J. Solut. Chem..

[13] Huzinaga S., Andzelm J., Klobukowski M., Radzio-Audzelm E., Sakai Y., Tatewaki H. (1984). Gaussian Basis Sets for Molecular Calculations.

[14] Easton R., Giesen D., Welch A., Cramer C., Truhlar D. (1996). The MIDI! Basis Set for Quantum Mechanical Calculations of Molecular Geometries and Partial Charges. Theor. Chem. Acc..

[15] Lewars E. (2003). Computational Chemistry—Introduction to the Theory and Applications of Molecular and Quantum Mechanics.

[16] Young D. (2001). Computational Chemistry—A Practical Guide for Applying Techniques to Real-World Problems.

[17] Jensen F. (2007). Introduction to Computational Chemistry.

[18] Cramer C. (2004). Essentials of Computational Chemistry—Theories and Models.

[19] Peverati R., Truhlar D.G. (2014). Quest for a Universal Density Functional: The Accuracy of Density Functionals Across a Broad Spectrum of Databases in Chemistry and Physics. Philos. Trans. R. Soc. A.

[20] Glossman-Mitnik D. (2013). A Comparison of the Chemical Reactivity of Naringenin Calculated with M06 Family of Density Functionals. Chem. Cent. J..

[21] Martínez-Araya J.I., Salgado-Morán G., Glossman-Mitnik D. (2013). Computational Nanochemistry Report on the Oxicams—Conceptual DFT and Chemical Reactivity. J. Phys. Chem. B.

[22] Glossman-Mitnik D. (2013). Computational Nanochemistry Study of the Chemical Reactivity Properties of the Rhodamine B Molecule. Procedia Comput. Sci..

[23] Martínez-Araya J.I., Salgado-Morán G., Glossman-Mitnik D. (2013). Computational Nutraceutics: Chemical Reactivity Properties of the Flavonoid Naringin by Means of Conceptual DFT. J. Chem..

[24] Glossman-Mitnik D. (2014). Chemical Reactivity Theory within DFT Applied to the Study of the Prunin Flavonoid. Eur. Int. J. Sci. Technol..

[25] Glossman-Mitnik D. (2014). Computational Chemistry of Natural Products: A Comparison of the Chemical Reactivity of Isonaringin Calculated with the M06 Family of Density Functionals. J. Mol. Mod..

[26] Frau J., Muñoz F., Glossman-Mitnik D. (2016). A Molecular Electron Density Theory Study of the Chemical Reactivity of Cis- and Trans-Resveratrol. Molecules.

[27] Frau J., Glossman-Mitnik D. (2017). Chemical Reactivity Theory Study of Advanced Glycation Endproduct Inhibitors. Molecules.

[28] Parr R., Yang W. (1984). Density Functional Approach to the Frontier-Electron Theory of Chemical Reactivity. J. Am. Chem. Soc..

[29] Parr R., Szentpaly L., Liu S. (1999). Electrophilicity Index. J. Am. Chem. Soc..

[30] Morell C., Grand A., Toro-Labbé A. (2005). New Dual Descriptor for Chemical Reactivity. J. Phys. Chem. A.

[31] Morell C., Grand A., Toro-Labbé A. (2006). Theoretical Support for Using the Δ*f*(**r**) Descriptor. Chem. Phys. Lett..

[32] Cárdenas C., Rabi N., Ayers P., Morell C., Jaramillo P., Fuentealba P. (2009). Chemical Reactivity Descriptors for Ambiphilic Reagents: Dual Descriptor, Local Hypersoftness, and Electrostatic Potential. J. Phys. Chem. A.

[33] Ayers P., Morell C., de Proft F., Geerlings P. (2007). Understanding the Woodward-Hoffmann Rules by Using Changes in Electron Density. Chem. Eur. J..

[34] Morell C., Ayers P., Grand A., Gutiérrez-Oliva S., Toro-Labbé A. (2008). Rationalization of the Diels-Alder Reactions through the Use of the Dual Reactivity Descriptor Δ*f*(**r**). Phys. Chem. Chem. Phys..

[35] Morell C., Hocquet A., Grand A., Jamart-Grégoire B. (2008). A Conceptual DFT Study of Hydrazino Peptides: Assessment of the Nucleophilicity of the Nitrogen Atoms by Means of the Dual Descriptor Δ*f*(**r**). J. Mol. Struct. THEOCHEM.

[36] Martínez-Araya J.I. (2015). Why is the Dual Descriptor a More Accurate Local Reactivity Descriptor than Fukui Functions?. J. Math. Chem..

[37] Domingo L.R., Pérez P., Sáez J. (2013). Understanding the Local Reactivity in Polar Organic Reactions through Electrophilic and Nucleophilic Parr Functions. RSC Adv..

[38] Chamorro E., Pérez P., Domingo L.R. (2013). On the Nature of Parr Functions to Predict the Most Reactive Sites along Organic Polar Reactions. Chem. Phys. Lett..

[39] Frisch M.J., Trucks G.W., Schlegel H.B., Scuseria G.E., Robb M.A., Cheeseman J.R., Scalmani G., Barone V., Mennucci B., Petersson G.A. (2009). Gaussian 09 Revision D.01.

[40] Weigend F., Ahlrichs R. (2005). Balanced Basis Sets of Split Valence, Triple Zeta Valence and Quadruple Zeta Valence Quality for H to Rn: Design and Assessment of Accuracy. Phys. Chem. Chem. Phys..

[41] Weigend F. (2006). Accurate Coulomb-fitting Basis Sets for H to R. Phys. Chem. Chem. Phys..

[42] Peverati R., Truhlar D.G. (2011). Improving the Accuracy of Hybrid Meta-GGA Density Functionals by Range Separation. J. Phys. Chem. Lett..

[43] Peverati R., Truhlar D.G. (2012). M11-L: A Local Density Functional That Provides Improved Accuracy for Electronic Structure Calculations in Chemistry and Physics. J. Phys. Chem. Lett..

[44] Peverati R., Truhlar D.G. (2012). An Improved and Broadly Accurate Local Approximation to the Exchange-Correlation Density Functional: the MN12-L Functional for Electronic Structure Calculations in Chemistry and Physics. Phys. Chem. Chem. Phys..

[45] Peverati R., Truhlar D.G. (2012). Screened-Exchange Density Functionals with Broad Accuracy for Chemistry and Solid-State Physics. Phys. Chem. Chem. Phys..

[46] Peverati R., Truhlar D.G. (2012). Exchange-Correlation Functional with Good Accuracy for Both Structural and Energetic Properties while Depending Only on the Density and Its Gradient. J. Chem. Theory Comput..

[47] Marenich A., Cramer C., Truhlar D. (2009). Universal Solvation Model Based on Solute Electron Density and a Continuum Model of the Solvent Defined by the Bulk Dielectric Constant and Atomic Surface Tensions. J. Phys. Chem. B.

[48] Avogadro: An Open-Source Molecular Builder and Visualization Tool—Version 1.2.0. http://avogadro.openmolecules.net.

[49] Hanweel M., Curtis D.E., Lonie D., Vandermeersch T., Zurek E., Hutchison G. (2012). Avogadro: An Advanced Semantic Chemical Editor, Visualization, and Analysis Platform. J. Cheminf..

[50] Wang J., Wolf R.M., Caldwell J.W., Kollman P.A., Case D.A. (2004). Development and Testing of a General AMBER Force Field. J. Comp. Chem..

[51] Gorelsky S. (2011). AOMix Program for Molecular Orbital Analysis—Version 6.5.

[52] Gorelsky S., Lever A. (2001). Electronic Structure and Spectra of Ruthenium Diimine Complexes by Density Functional Theory and INDO/S - Comparison of the Two Methods. J. Organomet. Chem..

