# Supplementary material for: Computational Prediction of the Protonation Sites of Ac-Lys-(Ala)n-Lys-NH2 Peptides through Conceptual DFT Descriptors
Source: Molecules. 2017 Mar 13;22(3):458. doi: 10.3390/molecules22030458 (PMC6155279; doi:10.3390/molecules22030458)
Supplement: Supplementary file 1 [file molecules-22-00458-s001.pdf]

Computational Prediction of the Protonation Sites of Ac-Lys-(Ala)n-Lys-NH2 Peptides through Conceptual DFT Descriptors

Electronic Supplementary Information

Sebastián Sastre, Juan Frau and Daniel Glossman-Mitnik

Table S1A: HOMO and LUMO orbital energies (in eV), vertical ionization potentials I and electron affinities A (in eV), global electronegativity χ, total chemical hardness η, and global electrophilicity ω of KK, KAK, KA2K, KA3K, KA4K and KA5K peptides calculated with the M11 density functional and the Def2TZVP basis set using water as as solvent simulated with the SMD parametrization of the IEF-PCM model. The upper part of the table shows the results derived assuming the validity of the KID procedure and the lower part shows the results derived from the calculated ΔSCF vertical energies.

Property	HOMO	LUMO	χ_K	η_K	ω_K
KK	-9.450	1.922	3.764	11.372	0.623
KAK	-9.388	2.158	3.615	11.546	0.566
KA2K	-9.549	2.089	3.730	11.639	0.598
KA3K	-9.442	2.075	3.684	11.518	0.589
KA4K	-9.557	2.077	3.740	11.634	0.601
KA5K	-9.554	2.078	3.738	11.632	0.601
Property	I	A	χ	η	ω
KK	6.708	0.528	3.618	6.180	1.059
KAK	6.710	0.319	3.514	6.392	0.966
KA2K	6.695	0.270	3.482	6.425	0.944
KA3K	6.696	0.266	3.481	6.430	0.942
KA4K	6.702	0.248	3.475	6.453	0.936
KA5K	6.700	0.245	3.472	6.456	0.934

Table S1B: Descriptors J_I, J_A, J_{HL}, J_χ, J_η, J_ω and J_{D1} for the KK, KAK, KA2K, KA3K, KA4K and KA5K peptides calculated from the results of Table S1A

	J_I	J_A	J_{HL}	J_χ	J_η	J_ω	J_{D1}
KK	2.74	2.45	3.68	0.15	5.19	0.44	5.21
KAK	2.68	2.48	3.65	0.10	5.15	0.40	5.17
KA2K	2.85	2.36	3.70	0.25	5.21	0.35	5.23
KA3K	2.75	2.34	3.61	0.20	5.09	0.35	5.10
KA4K	2.86	2.32	3.68	0.27	5.18	0.33	5.20
KA5K	2.85	2.32	3.68	0.27	5.18	0.33	5.19
Average	2.79	2.38	3.67	0.20	5.17	0.37	5.19

Table S2A: HOMO and LUMO orbital energies (in eV), vertical ionization potentials I and electron affinities A (in eV), global electronegativity χ, total chemical hardness η, and global electrophilicity ω of KK, KAK, KA2K, KA3K, KA4K and KA5K peptides calculated with the M11L density functional and the Def2TZVP basis set using water as as solvent simulated with the SMD parametrization of the IEF-PCM model. The upper part of the table shows the results derived assuming the validity of the KID procedure and the lower part shows the results derived from the calculated ΔSCF vertical energies.

Property	HOMO	LUMO	χ_K	η_K	ω_K
KK	-6.135	-0.726	3.430	5.410	1.088
KAK	-6.153	-0.761	3.457	5.391	1.108
KA2K	-6.123	-0.811	3.467	5.312	1.132
KA3K	-6.126	-0.820	3.473	5.306	1.137
KA4K	-6.123	-0.828	3.475	5.295	1.140
KA5K	-6.123	-0.838	3.480	5.285	1.146
Property	I	A	χ	η	ω
KK	6.510	0.567	3.539	5.943	1.053
KAK	8.616	0.709	4.662	7.908	1.374
KA2K	8.240	0.770	4.505	7.470	1.358
KA3K	6.988	0.777	3.883	6.211	1.214
KA4K	8.203	0.782	4.492	7.421	1.360
KA5K	8.203	0.788	4.495	7.415	1.363

Table S2B: Descriptors J_I, J_A, J_{HL}, J_χ, J_η, J_ω and J_{D1} for the KK, KAK, KA2K, KA3K, KA4K and KA5K peptides calculated from the results of Table S2A

	J_I	J_A	J_{HL}	J_χ	J_η	J_ω	J_{D1}
KK	0.37	0.16	0.41	0.11	0.53	0.03	0.55
KAK	2.46	0.05	2.46	1.21	2.52	0.27	2.80
KA2K	2.12	0.04	2.12	1.04	2.16	0.23	2.41
KA3K	0.86	0.04	0.86	0.41	0.91	0.08	1.00
KA4K	2.08	0.05	2.08	1.02	2.13	0.22	2.37
KA5K	2.08	0.05	2.08	1.02	2.13	0.22	2.37
Average	1.66	0.07	1.67	0.80	1.73	0.17	1.91

Table S3A: HOMO and LUMO orbital energies (in eV), vertical ionization potentials I and electron affinities A (in eV), global electronegativity χ, total chemical hardness η, and global electrophilicity ω of KK, KAK, KA2K, KA3K, KA4K and KA5K peptides calculated with the MN12L density functional and the Def2TZVP basis set using water as as solvent simulated with the SMD parametrization of the IEF-PCM model. The upper part of the table shows the results derived assuming the validity of the KID procedure and the lower part shows the results derived from the calculated ΔSCF vertical energies.

Property	HOMO	LUMO	χ_K	η_K	ω_K
KK	-5.994	-0.486	3.240	5.508	0.853
KAK	-5.999	-0.335	3.167	5.664	0.886
KA2K	-6.030	-0.359	3.195	5.671	0.900
KA3K	-5.995	-0.357	3.176	5.638	0.894
KA4K	-6.029	-0.376	3.203	5.654	0.907
KA5K	-6.034	-0.369	3.201	5.665	0.905
Property	I	A	χ	η	ω
KK	8.294	0.280	4.287	8.015	1.147
KAK	7.557	0.185	3.871	7.372	1.017
KA2K	8.375	0.222	4.299	8.153	1.133
KA3K	8.280	0.231	4.255	8.049	1.125
KA4K	6.355	0.262	3.309	6.093	0.898
KA5K	8.448	0.261	4.354	8.187	1.158

Table S3B: Descriptors J_I, J_A, J_{HL}, J_χ, J_η, J_ω and J_{D1} for the KK, KAK, KA2K, KA3K, KA4K and KA5K peptides calculated from the results of Table S3A

	J_I	J_A	J_{HL}	J_χ	J_η	J_ω	J_{D1}
KK	2.30	0.21	2.31	1.05	2.51	0.19	2.72
KAK	1.56	0.15	1.57	0.70	1.71	0.13	1.85
KA2K	2.35	0.14	2.35	1.10	2.48	0.23	2.73
KA3K	2.28	0.13	2.29	1.08	2.41	0.23	2.65
KA4K	0.33	0.11	0.35	0.11	0.44	0.01	0.45
KA5K	2.41	0.11	2.42	1.15	2.52	0.25	2.78
Average	1.87	0.14	1.88	0.87	2.01	0.18	2.20

Table S4A: HOMO and LUMO orbital energies (in eV), vertical ionization potentials I and electron affinities A (in eV), global electronegativity χ, total chemical hardness η, and global electrophilicity ω of KK, KAK, KA2K, KA3K, KA4K and KA5K peptides calculated with the MN12SX density functional and the Def2TZVP basis set using water as as solvent simulated with the SMD parametrization of the IEF-PCM model. The upper part of the table shows the results derived assuming the validity of the KID procedure and the lower part shows the results derived from the calculated ΔSCF vertical energies.

Property	HOMO	LUMO	χ_K	η_K	ω_K
KK	-6.727	-0.291	3.509	6.437	0.956
KAK	-6.733	-0.389	3.561	6.344	0.999
KA2K	-6.782	-0.436	3.609	6.346	1.026
KA3K	-6.729	-0.434	3.582	6.295	1.019
KA4K	-6.783	-0.444	3.613	6.338	1.030
KA5K	-6.785	-0.454	3.619	6.331	1.035
Property	I	A	χ	η	ω
KK	6.622	0.355	3.488	6.267	0.971
KAK	6.628	0.338	3.483	6.290	0.965
KA2K	6.636	0.347	3.492	6.289	0.969
KA3K	6.624	0.333	3.478	6.291	0.962
KA4K	6.637	0.354	3.496	6.283	0.972
KA5K	6.639	0.354	3.497	6.285	0.973

Table S4B: Descriptors J_I, J_A, J_{HL}, J_χ, J_η, J_ω and J_{D1} for the KK, KAK, KA2K, KA3K, KA4K and KA5K peptides calculated from the results of Table S4A

	J_I	J_A	J_{HL}	J_χ	J_η	J_ω	J_{D1}
KK	0.11	0.06	0.12	0.02	0.17	0.01	0.17
KAK	0.11	0.05	0.12	0.08	0.05	0.04	0.10
KA2K	0.15	0.09	0.17	0.12	0.06	0.06	0.14
KA3K	0.11	0.10	0.15	0.10	0.00	0.06	0.12
KA4K	0.15	0.09	0.17	0.12	0.05	0.06	0.14
KA5K	0.15	0.10	0.18	0.12	0.05	0.06	0.14
Average	0.13	0.08	0.15	0.09	0.06	0.05	0.14

Table S5A: HOMO and LUMO orbital energies (in eV), vertical ionization potentials I and electron affinities A (in eV), global electronegativity χ, total chemical hardness η, and global electrophilicity ω of KK, KAK, KA2K, KA3K, KA4K and KA5K peptides calculated with the N12 density functional and the Def2TZVP basis set using water as as solvent simulated with the SMD parametrization of the IEF-PCM model. The upper part of the table shows the results derived assuming the validity of the KID procedure and the lower part shows the results derived from the calculated ΔSCF vertical energies.

Property	HOMO	LUMO	χ_K	η_K	ω_K
KK	-5.287	-0.775	3.031	4.512	1.018
KAK	-5.291	-0.731	3.011	4.560	0.994
KA2K	-5.298	-0.766	3.032	4.532	1.014
KA3K	-5.289	-0.753	3.021	4.536	1.006
KA4K	-5.297	-0.771	3.034	4.525	1.017
KA5K	-5.298	-0.768	3.033	4.530	1.016
Property	I	A	χ	η	ω
KK	5.675	0.404	3.039	5.272	0.876
KAK	6.673	0.473	3.573	6.200	1.030
KA2K	7.166	0.557	3.862	6.609	1.128
KA3K	5.955	0.575	3.265	5.380	0.991
KA4K	7.323	0.611	3.967	6.712	1.172
KA5K	7.368	0.622	3.995	6.746	1.183

Table S5B: Descriptors J_I, J_A, J_{HL}, J_χ, J_η, J_ω and J_{D1} for the KK, KAK, KA2K, KA3K, KA4K and KA5K peptides calculated from the results of Table S5A

	J_I	J_A	J_{HL}	J_χ	J_η	J_ω	J_{D1}
KK	0.39	0.37	0.54	0.01	0.76	0.14	0.77
KAK	1.38	0.26	1.41	0.56	1.64	0.04	1.73
KA2K	1.87	0.21	1.88	0.83	2.08	0.11	2.24
KA3K	0.67	0.18	0.69	0.24	0.84	0.02	0.88
KA4K	2.03	0.16	2.03	0.93	2.19	0.16	2.38
KA5K	2.07	0.15	2.08	0.96	2.22	0.17	2.42
Average	1.40	0.22	1.44	0.59	1.62	0.10	1.74

Table S6A: HOMO and LUMO orbital energies (in eV), vertical ionization potentials I and electron affinities A (in eV), global electronegativity χ, total chemical hardness η, and global electrophilicity ω of KK, KAK, KA2K, KA3K, KA4K and KA5K peptides calculated with the N12SX density functional and the Def2TZVP basis set using water as as solvent simulated with the SMD parametrization of the IEF-PCM model. The upper part of the table shows the results derived assuming the validity of the KID procedure and the lower part shows the results derived from the calculated ΔSCF vertical energies.

Property	HOMO	LUMO	χ_K	η_K	ω_K
KK	-6.361	-0.201	3.281	6.160	0.874
KAK	-6.365	-0.185	3.275	6.180	0.868
KA2K	-6.394	-0.182	3.288	6.212	0.870
KA3K	-6.362	-0.188	3.275	6.174	0.868
KA4K	-6.389	-0.198	3.294	6.190	0.876
KA5K	-6.393	-0.177	3.285	6.215	0.868
Property	I	A	χ	η	ω
KK	6.359	0.280	3.320	6.079	0.906
KAK	6.364	0.258	3.311	6.105	0.898
KA2K	6.395	0.245	3.320	6.150	0.896
KA3K	6.361	0.246	3.303	6.115	0.892
KA4K	6.383	0.252	3.318	6.131	0.898
KA5K	6.387	0.241	3.314	6.146	0.894

Table S6B: Descriptors J_I, J_A, J_{HL}, J_χ, J_η, J_ω and J_{D1} for the KK, KAK, KA2K, KA3K, KA4K and KA5K peptides calculated from the results of Table S6A

	J_I	J_A	J_{HL}	J_χ	J_η	J_ω	J_{D1}
KK	0.00	0.08	0.08	0.04	0.08	0.03	0.10
KAK	0.00	0.08	0.07	0.04	0.07	0.03	0.09
KA2K	0.00	0.07	0.06	0.03	0.06	0.03	0.07
KA3K	0.00	0.06	0.06	0.03	0.06	0.02	0.07
KA4K	0.01	0.05	0.05	0.02	0.06	0.02	0.07
KA5K	0.01	0.06	0.06	0.03	0.07	0.03	0.08
Average	0.00	0.07	0.07	0.03	0.07	0.03	0.08
